# Defective high‐density lipoprotein lipoprotection in type 2 diabetes during acute myocardial infarction is rescued by apolipoprotein M/sphingosine‐1‐phosphate loading

**DOI:** 10.1111/dom.70409

**Published:** 2025-12-28

**Authors:** Jens Vogt, Marcel Benkhoff, Nathalie H. Schröder, Hao Hu, Lisa Dannenberg, Petra Keul, Theresia Sarabhai, Kálmán Benedikt Bódis, Tobias Zeus, Philipp Wollnitzke, Malte Kelm, Amin Polzin, Bodo Levkau

**Affiliations:** ^1^ Institute of Molecular Medicine III, University Hospital Düsseldorf Heinrich Heine University Düsseldorf Düsseldorf Germany; ^2^ Department of Cardiology, Pulmonology, and Vascular Medicine Medical Faculty of the Heinrich Heine University Düsseldorf Düsseldorf Germany; ^3^ Cardiovascular Research Institute Düsseldorf (CARID) Düsseldorf Germany; ^4^ Department of Endocrinology and Diabetology, Medical Faculty and University Hospital Düsseldorf, Heinrich‐Heine‐University Düsseldorf Germany

## Abstract

**Aims:**

Mortality of patients with type 2 diabetes (T2D) after acute myocardial infarction (AMI) is tremendous and massively increased compared to non‐T2D individuals. The reasons are unclear. High‐density lipoprotein (HDL) conducts lipoprotection during AMI, leading to improved outcomes. We hypothesised that T2D‐HDL lacks lipoprotective properties.

**Materials and Methods:**

HDL was isolated from healthy, non‐T2D individuals and T2D patients. This human HDL was administered to mice undergoing AMI. Infarct size and cardiac function were assessed by histology and echocardiography. HDL composition was determined by mass spectrometry.

**Results:**

HDL from healthy individuals but not from T2D patients reduced infarct size and improved cardiac function after AMI. Analysing HDL composition revealed lack of sphingosine‐1‐phosphate and Apolipoprotein M in T2D‐HDL. Ex‐vivo loading of HDL with these components rescued T2D‐HDL lipoprotection.

**Conclusions:**

We revealed that T2D‐HDL lacks lipoprotection during AMI. This might be a reason for enhanced mortality of T2D patients after AMI. Furthermore, we found an option to rescue T2D‐HDL lipoprotection.

## 1 INTRODUCTION

Mortality of patients with type 2 diabetes (T2D) during acute myocardial infarction (AMI) is very high.^1^ The reasons for this are probably manifold but mostly unclear. Chronic inflammation in T2D certainly plays a role within this context.^2^ High‐density lipoprotein (HDL) derived markers are associated with increased inflammatory burden in metabolic diseases.^3^, ^4^ The uric acid/HDL‐ratio (UHR) is a prime example of this. In recent years, it has been shown that UHR is a strong predictor for several T2D‐related disease states like prediabetes, diabetic retinopathy, diabetic kidney disease or even cardiometabolic risk in T2D patients.^5^, ^6^, ^7^, ^8^, ^9^, ^10^, ^11^ Moreover, HDL is known to improve outcomes after AMI.^12^ Despite its clearly demonstrated lipoprotective effects, none of the trials aimed at raising HDL concentrations succeeded in improving patient outcomes.^13^ Notably, a big proportion of the included patients had diabetes. The REVEAL study included 37% of patients with diabetes,^14^ in ILLUMINATE 44% of the included patients had diabetes^15^ and in ACCELERATE the percentage of diabetes patients was 68%.^16^ This appears relevant because some defects in T2D‐HDL are already known. Recent studies described changes in the composition of the lipidome and proteome as well as oxidation and glycation of HDL‐associated proteins.^17^ This leads to altered functions such as reduced cholesterol‐efflux capacity and impaired anti‐oxidative and anti‐inflammatory efficiency.^18^ The functional mediators of HDL include a variety of biological entities. Many of these are responsible for more than one HDL function. The Apolipoprotein M (ApoM)/sphingosine‐1‐phosphate (S1P) axis, for example, mediates a broad variety of HDL functions.^19^, ^20^ Based on this, we hypothesised that HDL in T2D patients is dysfunctional and fails to provide effective lipoprotection during AMI. Hence, we aimed to characterise lipoprotective properties of T2D‐HDL during AMI, T2D‐HDL composition and tested options to restore T2D‐HDL lipoprotection.

## 2 MATERIALS AND METHODS

### 2.1 Research design

**FIGURE 1:**
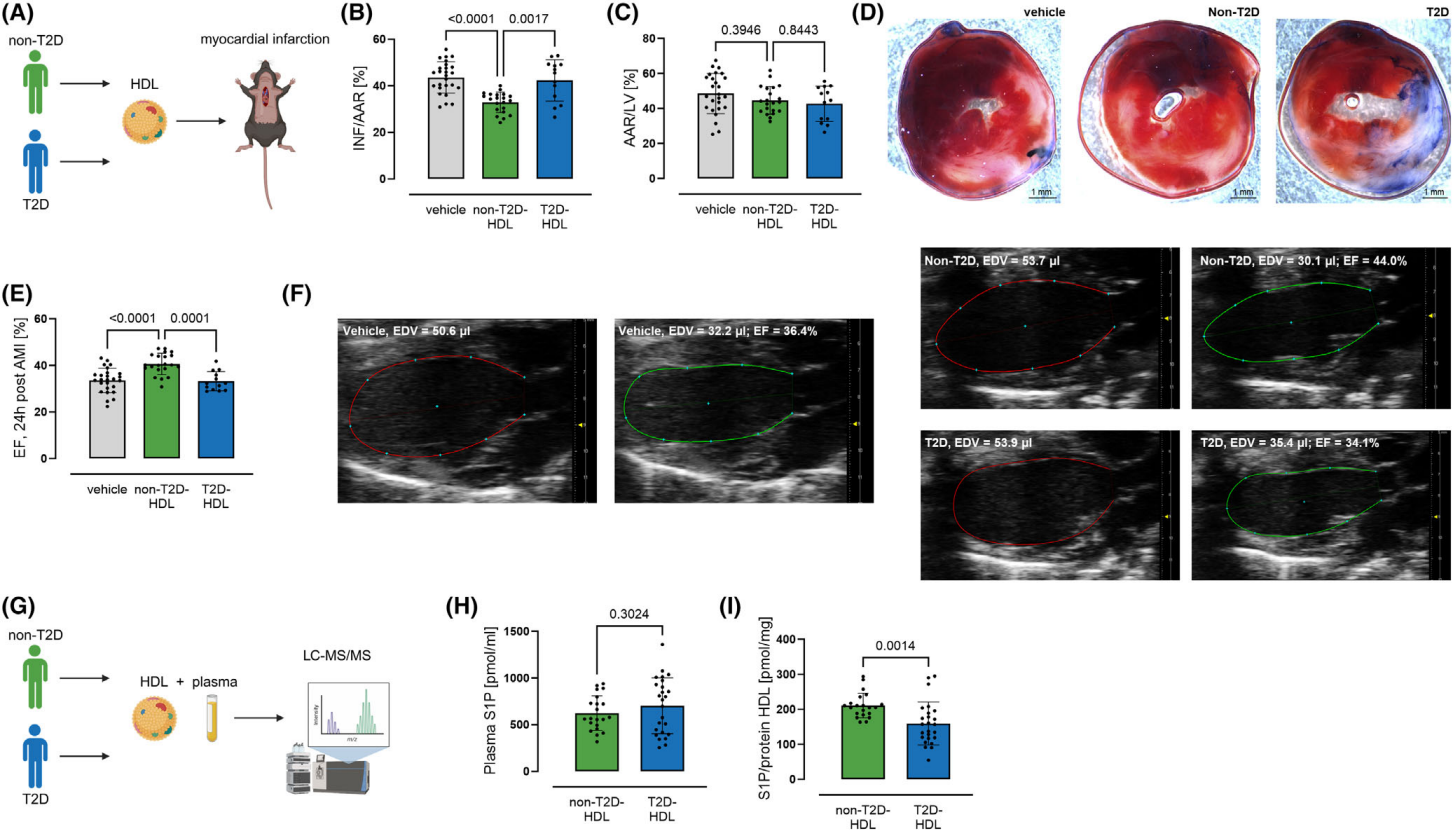
HDL from patients with type 2 diabetes showed defective cardioprotection. (A) HDL was isolated from plasma of healthy, non‐T2D volunteers or type 2 diabetes (T2D) patients by density ultracentrifugation and injected (43 mg HDL protein/kg) in the tail vein of C57Bl/6J mice 5 min prior to 30 min of ischemia. (B) Infarct size 24 h after murine coronary ischemia/reperfusion injury (INF/AAR; AAR = area at risk) of mice treated with non‐T2D‐HDL was reduced. However, T2D‐HDL showed no cardioprotection. (C) Area at risk did not differ between the groups. (D) Exemplary images of TTC‐stained heart section are provided. (E) Ejection fraction of these mice 24 h post AMI, determined by echocardiography, confirmed these findings (*n*_vehicle_ = 26, *n*_nonT2D‐HDL_ = 21, *n*_T2D‐HDL_ = 13, one‐way ANOVA followed by Tukey's multiple comparison test). (F) Exemplary, automatically determined LV‐Traces are provided (Vevo Lab, EDV = end‐diastolic volume, ESV = end‐systolic volume). (G) Sphingosine‐1‐phosphate (S1P) content of plasma and HDL was determined by LC–MS/MS. (H) Plasma S1P was unchanged between T2D patients and non‐T2D subject. (I) In contrast, HDL‐S1P of T2D patients was reduced compared to non‐T2D subjects (*n*_nonT2D‐HDL_ = 21, *n*_T2D‐HDL_ = 25, unpaired *t* test). Data are expressed as mean ± SD.

Cardioprotective properties of HDL were investigated by transfer experiments (Figure 1A). Therefore, HDL was isolated from healthy, non‐T2D individuals and T2D patients via ultracentrifugation. This human HDL was administered to mice undergoing experimental AMI. Cardioprotection was determined by assessment of infarct size and cardiac function. Next, HDL composition was analysed via liquid chromatography ‐ tandem mass spectrometry (LC–MS/MS) and enzyme‐linked immunosorbent assay (ELISA). Finally, isolated HDL from T2D patients was modified ex vivo. ApoM and S1P were loaded onto T2D‐HDL and administered to mice undergoing experimental AMI.

### 2.2 Human studies

The study was in accordance with the Declaration of Helsinki and was approved by the University of Düsseldorf Ethics Committee (vote no° 4658). People over the age of 18 who provided written informed consent were included. Individuals in the non‐T2D group had no cardiovascular disease and no history of T2D. Only people with a confirmed diagnosis of T2D were included in the T2D group. Exclusion criteria were a recent AMI or stroke in the last 6 months, current oncological diseases, and liver, kidney, and thyroid dysfunction. Characteristics of included persons are provided in Table S1, Supporting Information.

### 2.3 HDL isolation and loading

HDL was isolated from individual human plasma samples as previously described.^21^ In brief, HDL was isolated using a procedure to separate major lipoprotein classes from plasma by sequential density gradient ultracentrifugation using KBr solutions varying in density. After that, an HDL fraction was separated in the density range of 1.063 g/mL < d < 1.21 g/mL. Removal of KBr from the HDL samples was achieved via dialysis for 24 h at 4°C against four changes of at least 100‐fold volume of Ringer solution (Fresenius Kabi) each. Protein concentration of the HDL samples was determined with a Bradford assay (Bio‐Rad Laboratories). ApoM loading of HDL, if required, was conducted by co‐incubation of HDL with 40 μg ApoM (Sino Biological) per mg HDL at 4°C for 18 h. Unincorporated ApoM was removed by using centrifugal filters with a nominal molecular weight limit of 100 000 kDa (Merck). After that, incorporated ApoM content was quantitatively assessed by ELISA. S1P loading of HDL was performed by co‐incubation. For this, a corresponding calculated amount S1P solution for the HDL sample was transferred to a reaction tube and the solvent (methanol) was almost completely evaporated before HDL was added. Incubation time was 18 h at 4°C. Unbound S1P was removed by dialysis.

### 2.4 LC–MS/MS for S1P

S1P measurements were performed as described previously.^22^ In brief, chromatographic separation for S1P was performed with a 2 × 60 mm MultoHigh‐C18 RP column with 3 μm particle size at 40°C on a LCMS‐8050 triple quadrupole mass spectrometer (Shimadzu) interfaced with a Dual Ion Source and a Nexera X3 Front‐End‐System (Shimadzu). MS settings were the following: Interface: electrospray ionisation, nebulising gas flow: 3 L/min, heating gas flow: 10 L/min, interface temperature: 300°C, desolvation temperature: 526°C, desolvation line (DL) temperature: 250°C, heat block temperature: 400°C, drying gas flow: 10 L/min. Flow rate was 0.4 mL/min. Mobile phases for S1P measurement consisted of [A] = methanol and [B] = aqueous HCO_2_H (1% vol./vol.) and the following gradient settings were used: [A] increased from 10% to 100% over 3 min (B.curve = −2) and returned to 10% from 8.01 to 10 min prior to the next injection. Data were collected using multiple reaction monitoring (MRM) and positive ionisation was used for qualitative analysis and quantification. Standard curves were generated by measuring increased amounts of analytes with internal standard. All MS analyses were performed with LabSolutions 5.114, analysed with LabSolutions Insight (Shimadzu).

### 2.5 Determination of apolipoprotein M

ApoM content of HDL was measured via ELISA. Sample preparation was conducted according to the manufacturer's recommendation (Biozol).

### 2.6 Animal studies

C57BL/6J wild‐type mice were obtained from Janvier Labs (Saint‐Berthevin, France). All animals had unrestricted access to drinking water and standard chow. They were housed under controlled conditions, including a 12‐h light/dark cycle, constant temperature, and stable humidity. Myocardial infarction was induced, infarct size measured, and echocardiographic assessments performed as previously described.^23^, ^24^ HDL was injected at 43 mg HDL protein/kg body weight in the mouse tail vein in a volume of 150 μL 5 min prior to ischemia.^12^, ^25^ Each mouse was injected with HDL from a single donor. Vehicle control mice were treated with a solution of equimolar bovine serum albumin. All experimental procedures involving mice were conducted in accordance with the ARRIVE guidelines and were approved by the State Office for Nature, Environment and Consumer Protection (LANUV) of North Rhine‐Westphalia, Germany (file number 81‐02.04.2018.A143), in compliance with the European Convention for the Protection of Vertebrate Animals used for Experimental and Other Scientific Purposes. Unequal group sizes occurred due to randomised allocation to experimental groups and predefined exclusion criteria (e.g., unsuccessful ligation of left anterior descending artery (LAD)), leading to removal of individual animals in a non‐systematic manner. Blinded assessment was applied throughout, and statistical analyses (analysis of variance (ANOVA) and post‐hoc testing) were chosen for their robustness to unequal sample sizes.

### 2.7 Statistics

Data are expressed as mean ± SD. Statistical analysis was performed using GraphPad Prism 10 (GraphPad Software). To consider normality of distribution, the Kolmogorov–Smirnov test and D'Agostino‐Pearson test were used. Normally distributed continuous variables were analysed using *t*‐test; non‐normally distributed variables using Mann–Whitney *U* test. Statistical significance in a comparison of three or more groups was evaluated by a one‐ or two‐way ANOVA with multiple comparisons and Tukey's post‐hoc test. Differences in significances (*p* values) were considered significant at *p* < 0.05.

## 3 RESULTS

Cardioprotective properties of HDL were investigated by transfer experiments (Figure 1A). Human HDL from T2D patients and healthy, non‐T2D individuals were applied to mice undergoing AMI. The T2D patients were older (54.3 ± 17.1 vs. 30.7 ± 4.4 years) and mean body mass index was higher (31.9 ± 6.8 vs. 24.8 ± 2.8 kg/m^2^). They suffered more frequently from obesity (52% vs. 0%) and hypertension (64% vs. 0%). Accordingly, the medication differed, as the healthy, non‐T2D individuals did not take any significant medication. In contrast, 36% of the T2D used statins, 72% were on oral antidiabetic medication, and 12% used insulin. In addition, the T2D patients showed rather poor glycaemic control with an HbA_1c_ of 74.9 ± 20.0 mmol/mol (Table S1). HDL isolated from non‐T2D individuals significantly reduced infarct size and improved ejection fraction following AMI in mice. In contrast, HDL from patients with T2D did not show these protective effects (Figure 1B–F). Next, the content of cardioprotective sphingolipid S1P was measured in plasma and HDL via LC/MS (Figure 1G). While plasma S1P did not differ between non‐T2D individuals and T2D patients (Figure 1H), S1P content in HDL of T2D patients was reduced by 25% (Figure 1I). Therefore, T2D‐HDL may transfer its S1P to atherogenic lipoproteins such as very low density lipoprotein (VLDL), intermediate densitiy lipoprotein (IDL), and low‐density lipoprotein (LDL), which have been shown to acquire S1P from HDL.^26^

**FIGURE 2:**
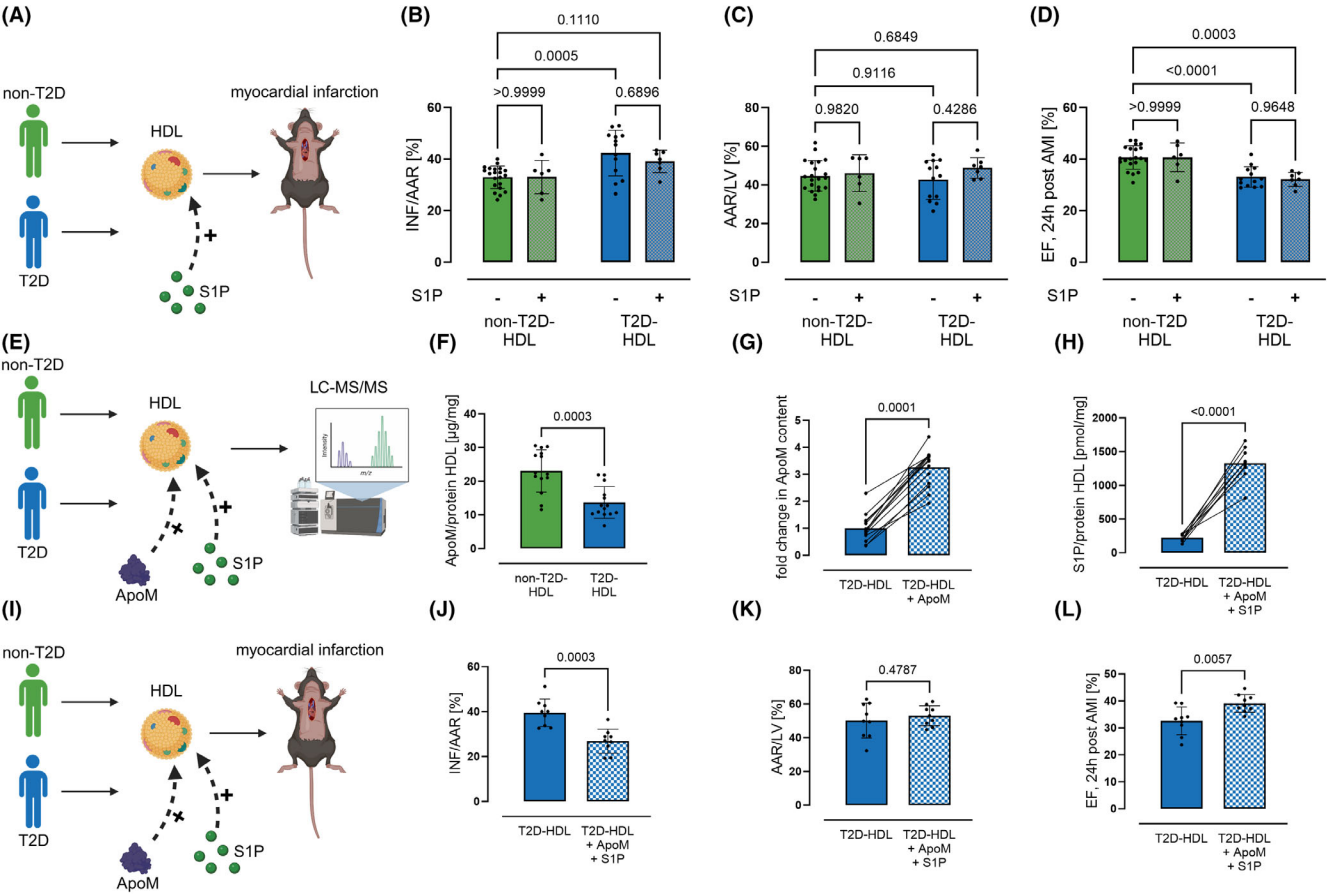
Loading with ApoM and S1P restored defective T2D‐HDL cardioprotection. (A) To restore cardioprotective effects, we loaded the HDL with S1P (38 μg S1P/kg in 43 mg HDL total protein/kg). (B–D) However, neither infarct size nor cardiac function were improved after S1P loading (*n*_nonT2D‐HDL_ = 21/6, *n*_T2D‐HDL_ = 13/7, two‐way ANOVA followed by Tukey's multiple comparison test). (E) T2D‐HDL was further analysed. (F) ApoM content was reduced in T2D‐HDL compared to HDL from non‐T2D individuals (*n*_nonT2D‐HDL_ = 14, *n*_T2D‐HDL_ = 14, Mann–Whitney *U* test). (G) To modify T2D‐HDL composition, ApoM was loaded to T2D‐HDL (*n*_nonT2D‐HDL_ = 14, *n*_T2D‐HDL_ = 14, paired *t* test). (H) After that, S1P loading of modified T2D‐HDL increased S1P content (*n*_nonT2D‐HDL_ = 9, *n*_T2D‐HDL_ = 9, paired *t* test). (I) Transfer experiments were conducted with modified T2D‐HDL in mice undergoing AMI. (J) ApoM and S1P loaded T2D‐HDL reduced the infarct size in mice 24 h after AMI, (K) whereas the area at risk did not differ between both groups. (L) Ejection fraction, measured 24 h after AMI, was improved in mice treated with T2D‐HDL loaded with ApoM and S1P (*n*_nonT2D‐HDL_ = 9, *n*_T2D‐HDL_ = 9, unpaired *t* test). Data are expressed as mean ± SD.

Hence, we aimed to restore the loss of HDL‐dependent cardioprotection by S1P‐loading (Figure 2A). However, S1P loading failed to improve the cardioprotective properties of T2D‐HDL (Figure 2B–D). The altered composition of HDL in T2D was then analysed further (Figure 2E). It was found that, in addition to the S1P reduction already shown, there was also a reduction in the S1P‐binding apolipoprotein M (ApoM, Figure 2F). We then tried to load the T2D‐HDL with ApoM in order to be able to successfully load S1P as well. As a result, we increased the ApoM content by a factor of 1.5 (Figure 2G). After that, we were successful in loading the T2D‐HDL with S1P (Figure 2H). Finally, we carried out further transfer experiments with this modified T2D‐HDL (Figure 2I). It was shown that the modification of the T2D‐HDL by loading with ApoM and S1P restored its lipoprotection (Figure 2J–L).

## 4 DISCUSSION

In our study, we (a) found blunted lipoprotection of T2D‐HDL; (b) demonstrated the mechanism by identifying loss of S1P and ApoM in T2D‐HDL; and (c) found a therapeutic approach to correct lipoprotection by T2D‐HDL.

HDL has clear lipoprotective effects. Additionally, high plasma concentrations of HDL are associated with decreased cardiovascular events.^27^, ^28^ Therefore, inhibitors of cholesteryl ester transfer protein were developed to massively increase HDL levels and thus reduce cardiovascular risk. However, this pharmacological approach failed to improve cardiovascular outcome despite a significant enhancement of HDL levels.^15^, ^16^ Even though the results are certainly based on multiple factors, this supports the hypothesis that HDL‐dependent lipoprotection is not dependent on quantity but on quality. This quality might be impaired in patients included in the trials. A closer analysis of the patient characteristics revealed that a large proportion of patients in all studies had metabolic disorders and diabetes.^15^, ^16^ We here conducted an analysis of HDL constitution and found impaired levels of S1P and ApoM in HDL of T2D patients. These findings align with previous analyses of T2D‐HDL.^29^, ^30^, ^31^ To minimise potential donor‐to‐donor variability in HDL composition that could have an impact on the results, each mouse received HDL from a single human donor. This design avoids pooling effects and allows the biological variability present in human HDL composition to be preserved and directly reflected in the experimental outcome. Moreover, the composition of all HDL samples was systematically characterised and these measurements confirmed the consistent reduction of S1P and ApoM across the T2D donor cohort. Thus, the key compositional defects underlying were not driven by isolated outliers but by a reproducible group‐level pattern. This underlines the hypothesis of impaired quality in these patients. In general, impaired HDL function in T2D is multifactorial. Next to reduced levels of S1P and ApoM, which we found in our analysis, other changes can also contribute to HDL dysfunction. Glycation of HDL led to impaired anti‐inflammatory and cholesterol efflux capacities.^32^ Moreover, glycation is also associated with decreased S1P content of HDL.^31^ Besides that, oxidative modifications occur in T2D‐HDL leading to diminished cholesterol efflux ability.^33^ Furthermore, the major anti‐oxidative agent of HDL, paraoxonase‐1 (PON1), showed reduced activity in oxidated HDL.^34^ Among other things, this led to reduced protection from oxidised LDL particles.^35^ However, it is not only the activity of PON1 that is altered in T2D, but also its quantity. Proteome analyses revealed changes in apolipoproteins and PON1.^30^ However, the role of ApoM in T2D is not entirely understood.^36^ It is known that HDL‐ApoM has important S1P‐independent functions such as stimulation of preβ‐HDL formation and cholesterol efflux,^19^ atheroprotection and potent anti‐oxidative properties.^20^ The bioactive sphingolipid S1P is one important mediator of HDL function.^37^ Especially, lipoprotection in AMI is achieved by S1P.^23^ This goes in line with the results of this study. This understanding could form the basis for targeted cardiovascular risk reduction in millions of people with T2D. Targeting HDL‐S1P may be a novel opportunity for tissue‐specific delivery of S1P or site‐specific S1P signalling. This is also promising because S1P signalling showed cardioprotection, no matter whether the S1P signalling was activated before, during, or after the infarction.^23^, ^38^, ^39^ HDL‐mediated tissue‐specific targeting of S1P signalling would be superior to other approaches such as pharmacological raising of S1P concentrations through S1P lyase inhibition or the use of S1P analogues such as fingolimod (Gilenya®). These approaches still harbour immunosuppressive side effects or cause bradycardia. For these reasons, S1P modulation in cardioprotection would be challenging. Accordingly, the currently preferred experimental mode of S1P administration in vivo has been to use manufactured “S1P chaperones” such as ApoM. This includes ApoM in the form of engineered fusion proteins (A*poM‐Fc*) or as part of reconstituted HDL preparations. Both approaches showed extended half‐life from minutes to days by protection from ectoenzymatic degradation.^40^ Indeed, ApoM‐associated HDL‐S1P and *ApoM‐Fc* were shown to protect the heart against I/R injury without inducing lymphopenia.^40^, ^41^ However, future studies will have to take several translational hurdles. Producing clinical‐grade ApoM (or ApoM‐Fc), S1P‐loaded HDL or donor‐derived HDL at scale with reproducible composition and safety is nontrivial. Nevertheless, understanding HDL‐S1P signalling may pave the way to exploiting new therapeutic approaches in cardiovascular and metabolic disease.

This study has some limitations. First, the composition of T2D‐HDL differs substantially from that of healthy individuals. In fact, levels of several dozen proteins and lipids are known to be changed.^30^ These changes especially occur in patients with poor glycaemic control and can lead to changes in the composition and fluidity of HDL affecting its function.^42^ Our T2D cohort showed rather poor glycaemic control. Therefore, changes in HDL composition and function might be more pronounced compared to HDL of patients with well‐controlled T2D. It is also possible that the loss of S1P is not the only reason for the loss of cardioprotection. Moreover, external loading with ApoM and S1P could alter the structure of HDL and thus also influence other HDL properties. However, it should be noted that regardless of all changes in the composition of T2D‐HDL, loading with ApoM+S1P led to a restoration of cardioprotection. Second, non‐T2D and T2D HDL donors differed regarding age, obesity and medication. Changes in HDL composition and function have been described for both obesity and age. Obesity markedly affects HDL metabolism, composition, and subclass distribution linked to changes in liver and adipose tissue.^43^ HDL of obese individuals showed a higher abundance of proteins related to inflammation. In contrast, anti‐oxidative and anti‐inflammatory proteins showed lower abundance.^44^ Moreover, lipidomic analyses of HDL revealed reduced levels of free cholesterol, cholesteryl‐esters, and phospholipids in obesity.^43^ The changes in HDL during aging are diverse and related to both lipids and proteins. HDL of elderly showed higher content of sphingomyelin, complement C3 and acute phase protein serum amyloid A whereas free cholesterol, ApoE and anti‐oxidative PON1 activity were reduced.^45^ In addition, increased HDL glycation occurred in elderly.^46^ These changes are associated with changes in HDL fluidity and function (cholesterol transport, antioxidation, etc.).^47^, ^48^ Medication, such as statins, can also have an impact on HDL.^49^ Even though the data available is not detailed, higher levels of ApoA1 and a shift to lipid‐rich HDL particles are known.^50^ Moreover, the expression of ApoM might be increased by some statins.^51^ With regard to T2D medication, it is difficult to distinguish between changes caused by the medication itself and changes that occur because the medication improves T2D and glycaemic control. Insulin does not appear to have any effect on HDL function.^52^ Current studies on SGLT2 inhibitors also showed no effect on HDL functionality.^53^ Other oral antidiabetic drugs show a slight improvement in HDL function, but this is associated with lower HDL glycation.^54^ However, to our knowledge, the cardioprotective properties of HDL in elderly or obese individuals have not been investigated. Our cohort is too small to highlight the influence of age, weight, and medication. Therefore, the possibility remains that these are confounders independent of T2D. Third, although well established, cross‐species transfer experiments may trigger immune or complement effects. However, the acute experimental timeframe, absence of histological signs of inflammation, and identical treatment conditions across T2D and non‐T2D group argue against differential immune or complement activation as an explanation for our findings. Fourth, interestingly we observed reduced HDL‐S1P despite preserved plasma S1P levels in T2D patients. Although our data suggest redistribution of S1P from HDL to apoB‐containing particles in T2D,^26^ we did not measure S1P content in individual VLDL, IDL, or LDL fractions. Therefore, the proposed shift of S1P towards apoB lipoproteins remains inferential and would benefit from direct validation in future studies.

## 5 CONCLUSION

In conclusion, we here found that T2D‐HDL lack S1P and ApoM, leading to blunted HDL‐dependent lipoprotection during AMI. We found an option to restore that by loading dysfunctional T2D‐HDL with ApoM and S1P. This needs to be tested in future analyses using different concentrations and time points before proceeding to large‐animal models. Finally, this could be a promising option to improve the impaired outcome of T2D patients with AMI.

## AUTHOR CONTRIBUTIONS

Malte Kelm, Amin Polzin and Bodo Levkau contributed to the conception of the work and co‐designed the study, analysed and interpreted data, and wrote the first draft of the paper with the input of Jens Vogt, Marcel Benkhoff, Nathalie H. Schröder, Lisa Dannenberg, and Philipp Wollnitzke. Theresia Sarabhai, Kálmán Benedikt Bódis, Tobias Zeus and Lisa Dannenberg managed the recruitment of the participants, patient data acquisition, analysed clinical data, and editing of the manuscript. Marcel Benkhoff and Hao Hu performed murine experiments including ligation operations, acquisition and interpretation of echocardiographic data and histological analyses, as well as data analysis, interpretation, figure preparation, and revision of the manuscript. Jens Vogt and Nathalie H. Schröder conducted HDL isolation, loading and all laboratory‐based HDL assays, and contributed to data collection, analysis, interpretation, preparation of figures, drafting, critical review, and editing the manuscript. Philipp Wollnitzke was responsible for and performed LC–MS/MS measurements and helped with the analysis of corresponding data. All of the authors have approved the final version to be published. Amin Polzin and Bodo Levkau are the guarantors of this work and take responsibility for the integrity of the data and the accuracy of the data analysis of the work as a whole.

## CONFLICT OF INTEREST STATEMENT

None.

## Supporting information


**Table S1:** Patient's characteristics. ACE = angiotensin converting enzyme, BMI = body mass index, HbA1_c_ = glycated haemoglobin, HDL = high‐density lipoprotein, LDL = low‐density lipoprotein, MI = myocardial infarction, PCI = percutaneous coronary intervention.

## Data Availability

Data are available from the corresponding author on reasonable request.
