# Supplementary material for: Defective high‐density lipoprotein lipoprotection in type 2 diabetes during acute myocardial infarction is rescued by apolipoprotein M/sphingosine‐1‐phosphate loading
Source: Diabetes Obes Metab. 2025 Dec 28;28(3):2175–82. doi: 10.1111/dom.70409 (PMC12890772; doi:10.1111/dom.70409)
Supplement: Supplementary file 1 — Table S1: Patient's characteristics. ACE = angiotensin converting enzyme, BMI = body mass index, HbA1c = glycated haemoglobin, HDL = high‐density lipoprotein, LDL = low‐density lipoprotein, MI = myocardial infarction, PCI = percutaneous coronary intervention. [file DOM-28-2175-s001.docx]

**Defective HDL Lipoprotection in Type 2 Diabetes During AMI is Rescued by ApoM/S1P Loading**

***Supplementary information***

***Supplementary table 1***

**Corresponding authors:**

Prof. Dr. med. Amin Polzin

Moorenstraße 5, 40225 Düsseldorf

Klinik für Kardiologie, Pneumologie und Angiologie

Universitätsklinikum Düsseldorf

Phone: 0049-211-18800

amin.polzin@med.uni-duesseldorf.de

Prof. Dr. med. Bodo Levkau

Institute of Molecular Medicine III

University Hospital Düsseldorf

Universitätsstr. 1

40225 Düsseldorf, Germany

Phone: 0049-211-18800

bodo.levkau@med.uni-duesseldorf.de

| Baseline characteristics | healthy, non-T2D  n=21 | T2D  n=25 | p-value |
| --- | --- | --- | --- |
| Male gender - no. (%) | 14 (66.6%) | 20 (80.0%) | .335 |
| Age - mean±SD | 30.7±4.4 | 54.3±17.1 | .001 |
| Weight - mean±SD | 80.5±11.9 | 100.5±26.0 | .001 |
| BMI -mean±SD | 24.8±2.8 | 31.9±6.8 | .001 |
| Obesity - no. (%) | 0 (0.0%) | 13 (52.0%) | .001 |
| Nicotine abuse - no. (%) | 3 (14.3%) | 5 (20.0%) | .710 |
| Triglycerides (mg/dl) | 136.4±34.9 | 162.4±80.1 | .175 |
| HDL (mg/dl) | 63.5±15.1 | 44.4±14.7 | .001 |
| LDL (mg/dl) | 118.0±32.9 | 91.1±37.0 | .001 |
| HbA_1c_ (mmol/mol) | / | 74.9±20.0 | / |
| HbA_1c_ (%) | / | 9.0±2.4 | / |
| Prior MI - no. (%) | 0 (0.0%) | 1 (4.0%) | .999 |
| Prior Stroke - no. (%) | 0 (0.0%) | 2 (8.0%) | .493 |
| Prior PCI - no. (%) | 0 (0.0%) | 2 (8.0%) | .493 |
| Hypertension - no. (%) | 0 (0.0%) | 16 (64.0%) | .001 |
| chronic kidney disease - no. (%) | 0 (0.0%) | 2 (8.0%) | .493 |
| coronary artery disease - no. (%) | 0 (0.0%) | 3 (12.0%) | .239 |
| Co-Medication – no. (%) |  | | |
| Aspirin | 0 (0.0%) | 6 (24.0%) | .025 |
| Oral anticoagulation | 0 (0.0%) | 0 (0.0%) | .999 |
| ACE inhibitors | 0 (0.0%) | 12 (46.0%) | .001 |
| Angiotensin Ⅱ receptor antagonists | 0 (0.0%) | 5 (20.0%) | .054 |
| Beta Blocker | 0 (0.0%) | 5 (20.0%) | .054 |
| Calcium antagonist | 0 (0.0%) | 6 (24.0%) | .025 |
| Proton pump inhibitor | 2 (9.5%) | 6 (24.0%) | .260 |
| Statin | 0 (0.0%) | 9 (36.0%) | .001 |
| Oral antidiabetics | 0 (0.0%) | 18 (72.0%) | .001 |
| Insulin | 0 (0.0%) | 12 (48.0%) | .001 |

**Table S1: Patient’s characteristics.** ACE = angiotensin converting enzyme, BMI = body mass index, HbA1_c_ = glycated hemoglobin, HDL = high-density lipoprotein, LDL = low-density lipoprotein, MI = myocardial infarction, PCI = percutaneous coronary intervention,
